# The complement receptor 3 (CD11b/CD18) agonist Leukadherin‐1 suppresses human innate inflammatory signalling

**DOI:** 10.1111/cei.12803

**Published:** 2016-07-26

**Authors:** A. L. Roberts, B. G. Fürnrohr, T. J. Vyse, B. Rhodes

**Affiliations:** ^1^ Division of Genetics and Molecular Medicine and Division of Infection, Immunity and Inflammatory DiseaseKing's College LondonLondonUK; ^2^ Division of Biological Chemistry, Innrain 80/IVMedical University InnsbruckInnsbruckAustria; ^3^ Department of RheumatologyUniversity Hospitals Birmingham NHS Foundation TrustBirminghamUK

**Keywords:** complement receptor 3, innate immunity, Leukadherin‐1, NK cells, systemic lupus erythematosus

## Abstract

Complement receptor 3 (CR3, CD11b/CD18) is a multi‐functional receptor expressed predominantly on myeloid and natural killer (NK) cells. The R77H variant of CD11b, encoded by the *ITGAM* rs1143679 polymorphism, is associated robustly with development of the autoimmune disease systemic lupus erythematosus (SLE) and impairs CR3 function, including its regulatory role on monocyte immune signalling. The role of CR3 in NK cell function is unknown. Leukadherin‐1 is a specific small‐molecule CR3 agonist that has shown therapeutic promise in animal models of vascular injury and inflammation. We show that Leukadherin‐1 pretreatment reduces secretion of interferon (IFN)‐γ, tumour necrosis factor (TNF) and macrophage inflammatory protein (MIP)‐1β by monokine‐stimulated NK cells. It was associated with a reduction in phosphorylated signal transducer and activator of transcription (pSTAT)‐5 following interleukin (IL)‐12 + IL‐15 stimulation (*P <* 0·02) and increased IL‐10 secretion following IL‐12 + IL‐18 stimulation (*P <* 0·001). Leukadherin‐1 pretreatment also reduces secretion of IL‐1β, IL‐6 and TNF by Toll‐like receptor (TLR)‐2 and TLR‐7/8‐stimulated monocytes (*P <* 0·01 for all). The R77H variant did not affect NK cell response to Leukadherin‐1 using *ex‐vivo* cells from homozygous donors; nor did the variant influence CR3 expression by these cell types, in contrast to a recent report. These data extend our understanding of CR3 biology by demonstrating that activation potently modifies innate immune inflammatory signalling, including a previously undocumented role in NK cell function. We discuss the potential relevance of this to the pathogenesis of SLE. Leukadherin‐1 appears to mediate its anti‐inflammatory effect irrespective of the SLE‐risk genotype of CR3, providing further evidence to support its evaluation of Leukadherin‐1 as a potential therapeutic for autoimmune disease.

## Introduction

Complement receptor 3 (CR3), also known as Mac1 or the αMβ2 integrin (CD11b/CD18), is a multi‐functional receptor expressed constitutively on neutrophils, myeloid cells and natural killer (NK) cells, as well as on subsets of activated lymphocytes. It binds a diverse range of ligands, which include iC3b (the stable, biologically active, solid‐phase degradation fragment of complement component C3), the intercellular adhesion molecule ICAM‐1 and the coagulation protein fibrinogen 1, 2. CR3 has long been recognized as a mediator of cell adhesion and phagocytosis, capable of triggering phagocytosis itself and also mediating phagocytosis following ligand engagement by glycophosphatidylinositol (GPI)‐linked FcγRIIIB (CD16b) on neutrophils 3. Recent studies demonstrate that CR3 also plays an important role in a broad range of immune signalling pathways. In particular, CR3 ligation suppresses inflammatory cytokine release following monocyte Toll‐like receptor (TLR) or interferon (IFN)‐α receptor activation, positively regulates TLR‐4 signalling in dendritic cells and regulates B cell receptor signalling 4, 5, 6, 7.

Two recent advances have increased our interest in the biology of CR3 signalling. First, a non‐synonymous (R77H) variant of the CR3 α‐chain, CD11b (encoded by the common rs1143679 variant of *ITGAM*), has been identified as a strong genetic risk factor for the development of the autoimmune disease systemic lupus erythematosus (SLE) across multiple populations [odds ratio (OR)_meta_ = 1.76] 8, 9, 10. This variant impairs CR3 function in neutrophils, monocytes and dendritic cells, but a detailed understanding of all the implications of genetically determined CR3 under‐functioning will be needed before we can begin to understand clearly the molecular pathways by which this disease susceptibility effect operates 11, 12, 13, 14. Secondly, a specific small‐molecule agonist of CR3, Leukadherin‐1, has been identified recently 15. Current data suggest that this drug mimics the effect of natural ligand in inhibiting monocyte TLR‐7/8‐induced tumour necrosis factor (TNF) secretion in a myeloid differentiation primary response gene 88 (MyD88)‐dependent manner 16. *In‐vivo* data suggest that this drug has potent anti‐inflammatory effects in a range of animal models, including an autoimmune nephritis model, without obvious short‐term side effects 17, 18. Leukadherin‐1 therefore appears to have therapeutic potential, and drug mechanisms with genetic support are estimated to succeed twice as often as those without it 19.

The NK cell is the only human cell type which constitutively expresses CR3 for which there are no published data on CR3 function. Therefore, the primary aim of our study was to use Leukadherin‐1 to explore how CR3 activation modifies NK cell cytokine release in response to innate immune stimuli, in a way that might be relevant to SLE disease mechanisms. A secondary aim was to broaden the existing published data on the influence of Leukadherin‐1 on monocyte signalling, which is important to understand in detail as part of preclinical evaluation of this potential therapeutic. To support the possibility of using Leukadherin‐1 to ‘overcome’ the genetic defect in CR3 function due to the R77H mutation we tested *ex‐vivo* cells from donors homozygous for the wild‐type or under‐functioning variant. Finally, using these genotyped cells we evaluated CR3 expression across a broad range of leucocyte subsets, thus refuting published data which suggests that genotype influences expression.

## Methods

### Study design

This experimental laboratory study used *ex‐vivo* leucocytes from healthy donors sourced from the Cambridge Bioresource and selected on the basis of the R77H CD11b variant, known from existing genotyping of the encoding rs1143679 *ITGAM* polymorphism. It was approved by the South East London Research Ethics committee and volunteers gave written informed consent. None of the volunteers had SLE or other systemic autoimmune disease and none were taking steroids or immune‐suppressing medication. When samples were obtained from a variant 77H homozygous donor these were paired with a wild‐type R77 homozygous donor and processed simultaneously, with efforts made to match pairs for age [mean ± standard deviation (s.d.) R77 donors 55·3 (13·4), 77H donors 48·2 (14·2)]. Laboratory investigators were blind to genotype, with genetic information released from the Cambridge Bioresource at analysis. All presented data are from R77 samples except where specified.

### Reagents

Fluorochrome‐conjugated antibodies were from eBiosciences (Hatfield, UK), anti‐CD210 (IL‐10R) and control from Biolegend (London, UK) [LEAF purified (low endotoxin, azide‐free)] and rabbit anti‐streptavidin antibodies from Abcam (Cambridge, UK). iC3b, Syk inhibitor IV and Leukadherin‐1 were from Calbiochem (Beeston, UK). Salt solutions and media were from Lifetech (Paisley, UK) except lymphocyte growth medium from Clonetics (Wokingham, UK). Polystyrene microspheres were from Spherotech (Sheffield, UK), IL‐12 and IL‐15 from Peprotech (London, UK), IL‐18 from R&D Systems (Abingdon, UK) and TLR agonists from Invivogen (San Diego, California, USA). Cytokines were quantified by cytometric bead array from BD Biosciences (San Jose, California, USA). Phosflow reagents were from BD Biosciences.

### Cell isolation

We used fresh blood with a maximum 4 h between bleed and cell purification. Peripheral blood mononuclear cells (PBMCs) were isolated by density gradient centrifugation and monocytes and NK cells subpurified by negative selection (Miltenyi Pan‐monocyte and NK cell isolation kits; Miltenyi Biotech, Bergisch Gladbach, Germany) and diluted to 7·5 × 10^5^/ml in serum‐free medium [macrophage serum‐free medium (monocytes) or lymphocyte growth medium (NK cells)]. The cell purity of each sample was assessed by flow cytometry: monocytes were characterized by their forward‐ and side‐scatter, positive expression of CD14 and negative expression of CD3, CD19 and CD56 and NK cells characterized by their forward‐ and side‐scatter, negative expression of CD3, CD19 and CD14 and positive expression of CD56. Samples with < 95% final purity or > 1% contamination by any single cell population were excluded from further analysis. Viability was assessed by trypan blue exclusion. Final isolates were 96% pure, 90% viable (NK cells) and 98% pure, 89% viable (monocytes).

### CD11b expression by flow cytometry

We used a multi‐colour flow‐cytometry panel (CD11b (or isotype control), CD3, CD14, CD16 and CD56) plus forward‐ and side‐scatter to evaluate the expression of CD11b on freshly isolated peripheral blood mononuclear cells following density gradient centrifugation. After exclusion of doublets, monocytes were identified by their characteristic forward‐ and side‐scatter and lack of CD3 and CD56 expression, and then subdivided on the bases of CD14 and CD16 expression. After exclusion of doublets, NK cells were identified by their forward‐ and side‐scatter (ensuring that the gate was set wide enough to capture the larger NK cells in the lymphocyte population), lack of CD3 and CD14 expression and positive expression of CD56.

### Cell stimulation

Supernatant cytokines were quantified after stimulation and culture for 18 h (monocytes) or 24 h (NK cells). Except for bead‐based stimulation, all experiments were conducted using 100 µl cells in a 96‐well plate format. NK cell stimuli (where used) were added as follows: (1) Syk inhibitor (1 μM), (2) Leukadherin‐1 or dimethylsulphoxide (DMSO) (vector control) (7·5 μM). Shown to induce ∼82% of maximum response with negligible off‐target effect 17), (3) anti‐CD210 or isotype control (5 µg/ml), (4) 30‐45 min after Leukadherin‐1 NK cells were stimulated with combinations of IL‐12 (10 ng/ml), IL‐15 (30 ng/ml) or IL‐18 (10 ng/ml): either IL‐12 + IL‐15 or IL‐12 + IL‐18. Monocytes were stimulated using pam3csk4 (TLR‐2 agonist, 300 ng/ml) or R848 (TLR‐7/8 agonist, 2 µg/ml). Supernatants were stored at −80ºC for < 1 month before quantification. To exclude non‐specific Leukadherin‐1‐mediated cytotoxicity we assessed cell viability at 24 h using the CellTitre‐Glo reagent (Promega, Southampton, UK). No significant loss of viability in comparison with the DMSO control was seen, concurring with published data in other cell types 17.

To stimulate cells through clustered Fc‐receptor engagement, 6·0–8·0 µm streptavidin‐coated polystyrene microspheres were incubated with 1 : 1000 polyclonal rabbit anti‐streptavidin immunoglobulin (Ig)G, followed by repeated washing. NK cells were treated initially with Leukadherin‐1 (or DMSO control) and incubated with end‐over‐end mixing for 30 min. Coated microspheres (1 : 8 cell : sphere ratio) were added with mixing for a further 1 h before static culture for 23 h.

### Cell signalling

To measure p38, extracellular‐regulated kinase (ERK), Syk and signal transducer and activator of transcription (STAT) phosphorylation, 2 × 10^5^ NK cells in 200 μl medium were stimulated as above. Non‐activated samples were included for fold‐change calculation. Cells were incubated at 37°C and fixed with 4% paraformaldehyde at specified time‐points. Fixed cells were surface‐stained with Pacific Blue‐conjugated anti‐CD56 for 45 min, permeabilized with PermBuffer III (BD Biosciences) on ice for 30 min, then stained with Alexa647‐conjugated phospho‐specific anti‐phosphorylated STAT (pSTAT)−3 (pY705), anti‐pSTAT‐4 (pY693), anti‐pSTAT‐5 (pY694), anti‐p‐p38 (pT180/Y182) or anti‐ERK1/2 (pT202/pY204) (BD Biosciences) for 1 h. Samples were run on a BDCanto II Flow cytometer and analysed with FlowJo software version 7·6·4 (TreeStar Inc., Ashland, OR, USA).

### Statistical analysis

Normally distributed data sets were compared by two‐tailed Student's *t*‐test (a paired test for within‐individual comparisons and unpaired for between‐individual comparisons). Skewed data were compared by the Wilcoxon matched‐pairs test, as specified in Results.

## Results

### Leukadherin‐1 modulates NK cell cytokine secretion

**Figure 1:**
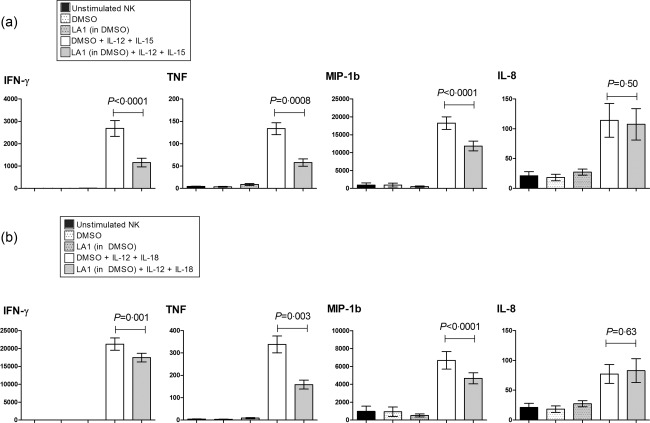
Leukadherin‐1 (LA1) modulates natural killer (NK) cell inflammatory cytokine secretion. Using cytometric bead array we showed that LA1 pretreatment reduced the interferon (IFN)‐γ, tumour necrosis factor (TNF) and macrophage inflammatory protein (MIP)‐1b release in primary NK cell cultures stimulated with (a) interleukin (IL)‐12+IL‐15 (*n* = 20) and (b) IL‐12+IL‐18 (*n* = 28). Dimethylsulphoxide (DMSO) provides the carrier control for LA1. Data presented are mean ± standard error of the supernatant cytokine concentration pg/ml at 24 h. Statistical testing by two‐tailed paired *t*‐test.

**Figure 2:**
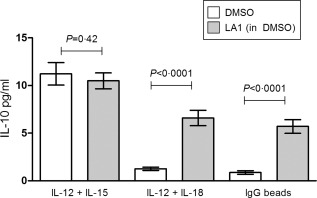
The effect of Leukadherin‐1 (LA1) on the secretion of interleukin (IL)−10 by natural killer (NK) cells. Using cytometric bead array we showed that LA1 pretreatment does not affect the IL‐10 production by cultured primary NK cells stimulated with IL‐12 + IL‐15 (*n* = 20). LA1 pretreatment significantly increases the IL‐10 production by primary NK cell cultures stimulated by IL‐12 + IL‐18 (*n* = 19) and immunoglobulin (Ig)G‐coated beads (*n* = 12). Dimethylsulphoxide (DMSO) provides the carrier control for Leukadherin. Data presented are median ± interquartile range of the supernatant cytokine concentration (pg/ml) at 24 h. Statistical testing by Wilcoxon's matched pairs test.

NK cells release a well‐documented repertoire of cytokines, including interferon‐γ (IFN‐γ), TNF, macrophage inflammatory protein‐1β [MIP‐1β (CCL4)] and interleukin‐8 [IL‐8 (CXCL8)], following activation by synergistic combinations of monokines 20. We observed a significant, large‐magnitude reduction in IFN‐γ, TNF and MIP‐1β secretion by monokine‐stimulated (IL‐12 + IL‐15, Fig. 1a; IL‐12 + IL‐18, Fig. 1b) NK cells that had been pretreated with Leukadherin‐1 (*P <* 0·001 for all; Fig. 1). No effect on IL‐8 secretion was observed following either monokine stimulations (*P* ≥ 0·5; Fig. 1), which demonstrates that Leukadherin‐1 does not globally suppress NK cell cytokine secretion. Unstimulated NK cells secreted only low levels of cytokine, with neither Leukadherin‐1 nor its DMSO vector, modifying this basal secretion significantly (Fig. 1). IL‐12 + IL‐15 stimulated cells secreted low levels of IL‐10 with no significant change seen with Leukadherin‐1 (Fig. 2). IL‐12 + IL‐18‐stimulated cells secreted barely detectable levels of IL‐10, but a small but significant increase was seen with Leukadherin‐1 (*P <* 0·0001; Fig. 2). Fc‐receptor engagement by clustered rabbit polyclonal IgG was sufficient to induce low‐level cytokine secretion without additional stimuli. Leukadherin‐1 did not modulate the secretion of IFN‐γ, TNF, MIP‐1β or IL‐8 under these conditions (Supporting information, Fig. S1), but did induce the secretion of small but significant amounts of IL‐10 (*P <* 0·0001; Fig. 2).

### The SLE‐associated CD11b‐R77H variant does not influence NK cell response to Leukadherin‐1

**Figure 3:**
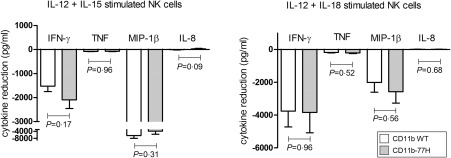
The systemic lupus erythematosus (SLE)‐associated CD11b‐R77H polymorphism does not affect the response of natural killer (NK) cells to Leukadherin‐1 (LA1). LA1 pretreated NK cells were compared for CD11b‐R77H genotype groups [wild‐type (WT) *versus* the rarer 77H variant]. Data presented as mean ± standard error reduction in cytokine secretion by NK cells consequent upon LA1 pretreatment. None of the observed differences are statistically significant (*P* > 0·09 for all).

The SLE‐associated CD11b‐R77H variant impairs CR3 function significantly in response to natural ligands 11, 12, 13, 14. We compared the Leukadherin‐1‐mediated reduction in inflammatory cytokine between homozygous WT (R77) and homozygous variant (77H) genotype groups (Fig. 3). We analysed the absolute change and the percentage change in cytokine secretion, using both unpaired *t*‐test to compare genotype groups and a paired *t*‐test for samples run in paired assays. Cytokine differences were not significant between genotype groups in any analysis (*P* > 0·09 for all).

### Leukadherin‐1 does not modulate Syk activation in NK cells

Monocyte studies implicate multiple mechanisms for CR3‐mediated modulation of inflammatory cytokine release, including Syk‐dependent inhibition and autocrine feedback by IL‐10 4, 5. We examined Syk‐signalling in NK cells and found that, in contrast to natural ligand‐engaged monocytes, Syk inhibition augmented rather than abrogated the inhibition of NK cell cytokine secretion seen with Leukadherin‐1 (Supporting information, Table S1). We also evaluated Syk activation by phosphorylation (pSyk) using Phosflow. After 30 min incubation with Leukadherin‐1 (or DMSO control) we observed no difference in NK pSyk fluorescence intensity (paired *t*‐test; total NK cells (*n* = 12) *P* = 0·53; CD56^hi^ NK cells (*n* = 11), *P* = 0·60, Supporting information, Fig. S2).

To evaluate whether IL‐10 autocrine feedback explained the effect of Leukadherin‐1 in NK cells we co‐incubated with anti‐CD210 (IL‐10 receptor) blocking antibodies. We observed no significant difference in cultured primary NK cell release of IFN‐γ, TNF, IL‐8, and MIP‐1β in the presence of anti‐CD210 antibody (Supporting information, Fig. S3).

### The effects of Leukadherin‐1 are STAT‐4‐independent

**Figure 4:**
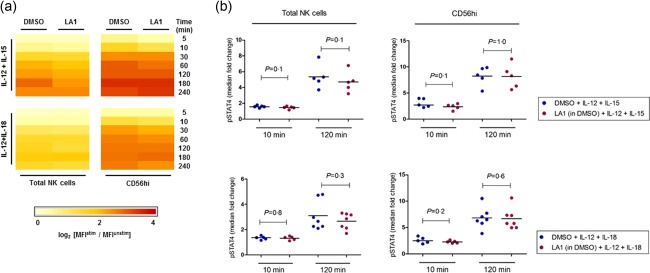
Leukadherin‐1 (LA1) does not modulate signal transducer and activator of transcription (STAT)‐4 phosphorylation. Using Phosflow intracellular flow cytometry assay we showed (a) the fold‐change increases in phosphorylated (p)STAT‐4 in total (left panel) and CD56^hi^ (right panel) natural killer (NK) cells, following IL‐12+IL‐15 (top panel) and IL‐12+IL‐18 (bottom panel) stimulation, is not modulated by LA1 pretreatment (data presented as log_2_ median fold‐change; 3 ≤ *n* ≤ 9 per time‐point). (b) No statistical difference was observed at early (10 min) or late (120 min) time‐points *(P* > 0·1 for all). Testing by Wilcoxon's matched‐pairs test. Dimethylsulphoxide (DMSO) provides the carrier control for LA1 in all panels.

To ascertain whether the Leukadherin‐1 effects were specific to IL‐12 signalling in both stimulation conditions (IL‐12 + IL‐15 and IL‐12 + IL‐18), we used PhosFlow to measure its effects on STAT‐4 phosphorylation across multiple time‐points ranging from 5 min to 4 h. Using cell surface staining of CD56 also enabled the simultaneous identification and analysis of the CD56^hi^ subset, which are the most efficient cytokine‐producing NK cell subset. As expected, we observed fold‐change increases in pSTAT‐4 following both IL‐12 + IL‐15 and IL‐12 + IL‐18 stimulation in both cell populations (Fig. 4a). No difference in median fold‐change of pSTAT‐4 was observed following Leukadherin‐1 pretreatment in either IL‐12 + IL‐15 or IL‐12 + IL‐18‐stimulated cells (*P* > 0·1 all; Fig. 4b).

### CR3 modulates IL‐15‐mediated STAT‐5 phosphorylation

**Figure 5:**
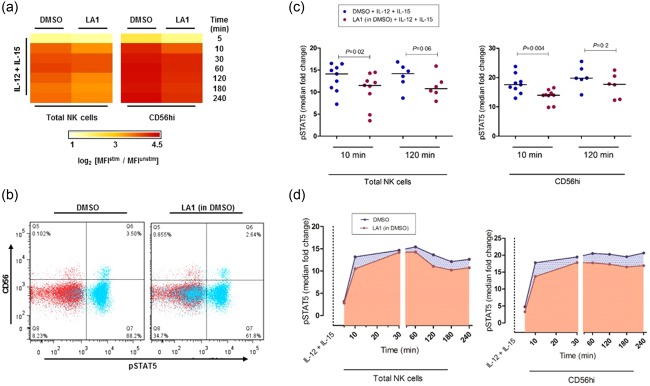
The effect of Leukadherin‐1 (LA1) on natural killer (NK) cell signal transducer and activator of transcription (STAT)‐5 phosphorylation. Using Phosflow intracellular flow cytometry assay we showed that LA1 pretreatment (a) reduced interleukin (IL)‐15‐mediated phosphorylated (p)STAT‐5 fold‐change in both total (left panel) and CD56^hi^ (right panel) NK cells across multiple time‐points (data presented as log_2_ median fold‐change; 3 ≤ *n* ≤ 9 per time‐point) and (b) reduced the percentage of pSTAT‐5‐positive cells (*x*‐axis) following IL‐12 + IL‐15 stimulation (blue). Basal levels of pSTAT‐5 are shown in red where no difference was observed. Representative data shown (*t* = 10min). (c) The median fold induction of pSTAT‐5 at 10 min was significantly lower in LA1‐treated cells in both total NK cells (left panel) and CD56^hi^ cells (right panel) (*n* = 9), and approached significance for both cell populations at 2 h (*n* = 6). Statistical testing by Wilcoxon's matched‐pairs test. (d) pSTAT‐5 fold‐change was observed to be consistently lower following LA1 pretreatment (red curve), during short‐ and long‐term IL‐12+IL‐15 stimulation in total NK cells (left panel) and the CD56^hi^ subset (right panel). Data presented as median fold‐change of pSTAT5 (3 ≤ *n* ≤ 9 per time‐point). Dimethylsulphoxide (DMSO) provides the carrier control for LA1 in all panels.

We next measured the effects of Leukadherin‐1 on the phosphorylation of intercellular signalling molecules known to be important in IL‐15 and IL‐18 signal transduction – mitogen‐activated protein kinase (MAPK) p38, ERK, STAT‐3 and STAT‐5 21, 22 – in stimulated NK cells across multiple time‐points. We observed a reduction in pSTAT‐5 at all time‐points (Fig. 5), both in the magnitude of pSTAT‐5 induction (fold‐change from time zero) (Fig. 5a,d) and in the proportion of pSTAT‐5‐positive cells (Fig. 5b), in IL‐12 + IL‐15‐stimulated cells pretreated with Leukadherin‐1. This difference was seen in both the total NK cells and the CD56^hi^ subpopulation. The effect is apparent from the earliest time‐point (5 min) and most significant at 10 min (Fig. 5c; total NK *P* = 0·02; CD56^hi^ *P* = 0·004), suggesting a mechanism of inhibited STAT‐5 phosphorylation rather than initiation of a dephosphorylation mechanism. No difference was observed in the median fold‐change of pSTAT‐5 in Leukadherin‐1 pretreated IL‐12 + IL‐18 cells (Supporting information, Fig. S4). Additionally, Leukadherin‐1 did not affect the median fold‐change of phosphorylation in MAPK p38, ERK, or STAT‐3 under either IL‐12 + IL‐15 or IL‐12 + IL‐18‐stimulated cells (Supporting information, Fig. S4).

### Leukadherin‐1 modulates TLR‐2 and TLR‐7/8‐induced monocyte cytokine secretion

**Figure 6:**
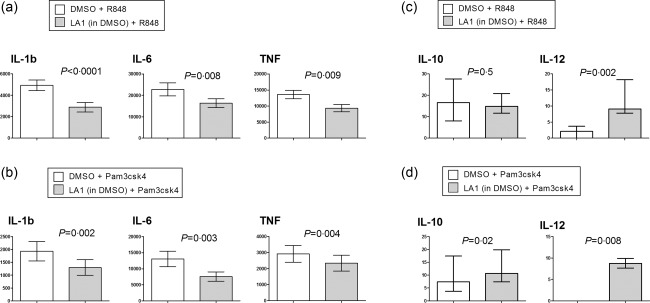
Leukadherin‐1 (LA1) modulates monocyte inflammatory cytokine secretion. Using cytometric bead array we showed that LA1 pretreatment reduced the interkeukin (IL)‐1b, IL‐6 and tumour necrosis factor (TNF) release in monocyte cultures stimulated with (a) Toll‐like receptor (TLR)‐7/8 agonist R848 (*n* = 18) and (b) TLR‐2 agonist Pam3csk4 (*n* = 11). We also showed that LA1 pretreatment (c) increased the IL‐12 release but did not affect the IL‐10 release by monocyte cultures stimulated with TLR‐7/8 agonist R848 (*n* = 18) and (d) increased the IL‐10 and IL‐12 release by monocyte cultures stimulated with TLR‐2 agonist Pam3csk4 (*n* = 11). Data presented in (a) and (b) are mean ± standard error of the supernatant concentration in pg/ml at 18 h, and statistical testing by paired *t*‐test. Data presented in (c) and (d) are median ± interquartile range of the supernatant cytokine concentration in pg/ml at 18 h, and statistical testing by Wilcoxon's matched‐pairs test. Dimethylsulphoxide (DMSO) provides the carrier control for LA1.

It has been reported previously that Leukadherin‐1 modulates the secretion of TNF secretion by monocytes following TLR‐7/8‐stimulation 16. We tested the hypothesis that Leukadherin‐1 modulates an entire cytokine repertoire following TLR‐7/8 stimulation and also modulates cytokine production in response to stimulation of TLR‐2. We observed a significant down‐regulation of the secretion of monocyte inflammatory cytokines interleukin‐1β (IL‐1β; *P <* 0·001) and interleukin‐6 (IL‐6; *P* = 0·008), in addition to TNF (*P* = 0·009), following TLR‐7/8 stimulation (Fig. 6a). We saw similar down‐regulation of TNF (*P* = 0·004), IL‐1β (*P* = 0·002), and IL‐6 (*P* = 0·003) secretion in TLR‐2‐stimulated monocytes (Fig. 6b).

IL‐10 was secreted at low levels by monocytes following either TLR‐2 (Fig. 6c) or TLR‐7/8 (Fig. 6d) stimulation, but was modified only weakly by Leukadherin‐1. Undetectable (TLR‐2 stimulation) or barely detectable (TLR‐7/8 stimulation) levels of IL‐12 were seen following TLR activation alone, with a very small but significant increase seen with the addition of Leukadherin‐1 (Fig. 6c, *P* = 0·002; Fig. 6d, *P* = 0·008).

### The CR3 R77H variant does not affect cell surface expression of CR3 on monocytes or NK cells

**Figure 7:**
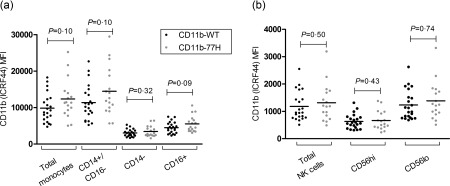
Systemic lupus erythematosus (SLE)‐associated R77H does not affect cell surface expression of CD11b on natural killer (NK) cells and monocytes. There was no difference in CD11b expression (anti‐CD11b fluorescence – isotype control) on primary monocytes and NK cells between CD11b‐R77H homozygote genotype groups [wild‐type (WT) *versus* 77H]. CD14 and CD16, and CD56 cell surface markers were used to identify monocyte and NK cell subsets, respectively. No genotypic difference was observed in any cell subset (*P* > 0·09 for all). Mean fluorescence intensity (MFI) for each donor is displayed. Statistical testing by unpaired *t*‐test.

A recent study suggested that the minor allele of the SLE‐associated rs1143679 (R77H) polymorphism is associated with reduced cell surface expression of CD11b on *ex‐vivo* monocytes 8. We used a multi‐colour flow‐cytometry panel as outlined in the Methods section to evaluate the expression of CD11b on monocyte and NK cell subsets between homozygous WT (R77) (*n* = 22) and homozygous variant (77H) (*n* = 17) genotype groups. In the monocyte population we compared four subgroups: total monocytes, CD14^hi^/CD16^–^ cells (classical monocytes), CD14^–^ (non‐classical monocytes) and also all CD16^+^ monocytes. As we observed previously in an earlier study, there was no significant difference (*P* = 0·1) in the cell surface expression of CD11b on any monocyte subset between the two homozygous genotype groups (Fig. 7) 12. In the NK cell population we also compared three subgroups: total NK cells, the small subset characterized by CD56^hi^ expression and the larger subset with regular CD56 expression only. Similarly, there was no significant difference in CD11b expression between the two genotype groups (*P* > 0·43 for all) (Fig. 7).

## Discussion

We have demonstrated that Leukadherin‐1 exerts a marked functional effect on the release of proinflammatory cytokines by monokine‐primed (IL‐12 + IL‐15/IL‐18) NK cells. Similar to previous reports in monocytes, this effect was broadly anti‐inflammatory. Whether this pathway contributes to the pathogenesis of SLE, and whether there is potential to manipulate this therapeutically using Leukadherin‐1, is an intriguing question.

Several lines of evidence implicate NK cells in the pathogenesis of SLE. For example, circulating NK cell numbers are reduced (probably migrated to lymphoid organs), but produce higher levels of interferon‐γ 23. NK cells up‐regulate the production of type 1 interferon (characteristically high in SLE) by co‐cultured plasmacytoid dendritic cells following stimulation by RNA‐containing lupus immune complexes 24. There is evidence from the B6.*Sle1* murine SLE model of an NK cell‐dependent, T cell‐independent pathway of autoantibody production, with lower anti‐nuclear antibody levels in NK‐depleted animals 25. Finally, there is a precedent for penetrant SLE genetic susceptibility effects (Fc‐receptor locus copy‐number variation) leading to changes in NK cell phenotype 26. We also know that monocytes are activated in SLE and produce monocyte‐derived cytokines (monokines). Both IL‐15 and IL‐18 are elevated in the serum of patients with lupus, along with an increase in leucocyte membrane‐bound IL‐15 27, 28. These monokines can trigger the early release of TNF and IFN‐γ from NK cells, thus perpetuating a proinflammatory milieu independent of, and most probably before, activation of the adaptive immune system.

Our results demonstrate that CR3 is a negative regulator of this inflammatory loop and that Leukadherin‐1 is capable of marked NK cell cytokine down‐regulation. Furthermore Leukadherin‐1 is equally capable of down‐regulating signalling even in cells that are homozygous for the genetically encoded under‐functioning variant of CR3, presumably because the mechanism of CR3 signal transduction is different following engagement with this small molecule compound compared with natural ligand. We did not demonstrate any significant effect of Leukadherin‐1 in modulating cytokine production following engagement of CD16 on NK cells. Whether this would also apply to other CD16‐mediated functions, such as antibody‐dependent cellular cytotoxicity (ADCC), would require further investigation.

In addition to documenting *ex‐vivo* effects of Leukadherin‐1 we have, in part, explained the mechanism in NK cells by demonstrating an inhibition of IL‐15‐induced phosphorylation of the transcription factor STAT‐5. Our data suggest that Leukadherin‐1 interferes with events that take place upstream of, or during, STAT‐5 phosphorylation – such as Janus kinase (JAK) phosphorylation or JAK/STAT association – rather than through a mechanism of STAT dephosphorylation. Activation of JAK‐1 and JAK‐3 associated with the IL‐2Rβ and γc‐chains, respectively, has been shown to be crucial for STAT‐5 tyrosine phosphorylation in response to IL‐15 22, 29. A CR3‐mediated effect on JAKs has been shown recently in human monocytes/macrophages, in which CR3 activation with anti‐CD18 or anti‐CD11b antibodies blocks the phosphorylation of IL‐13 receptor‐associated JAK‐2 and tyrosine kinase‐2 (TYK‐2) kinases, which further inhibits the downstream tyrosine and serine phosphorylation of STAT‐1, STAT‐3 and STAT‐6 30. Further work is needed to determine the upstream mechanism through which the Leukadherin‐1‐mediated inhibition of STAT‐5 phosphorylation occurs in NK cells.

We have also shown that the modulation of NK cells by Leukadherin‐1 is STAT‐4‐independent. *STAT4* polymorphisms are robustly associated with SLE 31, and our data suggest that the effects of Leukadherin‐1 will not be confounded by SLE‐associated *STAT4* polymorphisms. We have also discounted Syk‐activation and IL‐10 autocrine feedback, both of which are important mechanisms in monocytes following engagement by natural ligand. Clearly, pSTAT‐5 inhibition is not the only Leukadherin‐1 influenced pathway in NK cells, because IL‐12 + IL‐18 stimulation was not associated in any notable STAT‐5 phosphorylation: Leukadherin‐1 must operate differently under these conditions and this will require further experimental evaluation.

Having conducted a novel evaluation of NK cells, we then aimed to replicate and significantly extend existing data on the effect of Leukadherin‐1 in monocytes. We demonstrated a broad inhibition of proinflammatory cytokines (TNF, IL‐1β and IL‐6) following activation of both TLR‐7/8 and TLR‐2. TLR activation is recognized increasingly as an element of SLE pathogenesis. SLE is characterized by the production of high‐affinity antibodies that target nuclear protein/nucleic acid antigens (Ro, La, RNP and Sm) and anti‐dsDNA 32. These antigens function as immunological adjuvants by activating nucleic acid‐sensing TLR‐7/8 and ‐9 33, 34. The activation of monocyte TLR‐7/8 triggers the production of inflammatory cytokines (IL‐1β, IL‐6, TNF) and cytokines which augment the adaptive immune response (IL‐12, IL‐18) 34, 35. The activation of TLR‐2 by components of late‐apoptotic and secondary necrotic cells may be another inflammatory mechanism in SLE, where defective apoptotic cell clearance is well described 36. In particular, high mobility group box 1 (HMGB1) containing nucleosomes from apoptotic cells generate a TLR‐2‐dependent inflammatory response and, as a component of dsDNA‐containing immune complexes, induce TLR‐2‐dependent anti‐dsDNA antibody production 37, 38.

In addition, we have demonstrated that the SLE‐associated R77H variant does not affect the cell surface expression of CD11b on *ex‐vivo* monocytes and NK cells. A recent study by Maiti *et al*., in contradiction to other published reports, suggested a genotypic difference in CD11b expression on CD14 positively selected monocytes 8. We believe our data from freshly isolated peripheral blood mononuclear cells following density gradient centrifugation to be robust, with minimal confounding factors from positive selection of cell populations. Our comprehensive expression data demonstrate stark variation in CD11b across the three monocyte subsets, as shown previously for other cell surface receptors, but no genotype effect 39.

Genetic and functional studies implicate under‐functioning CR3 as a risk factor for SLE, suggesting intuitively that augmenting CR3 function may be an effective therapeutic approach 9, 10, 11, 12, 13, 14. Our data suggest that there is a potential to achieve this using a small‐molecule compound which, *ex‐vivo*, exerts broadly anti‐inflammatory effects on two innate immune cell types. In other autoimmune diseases innate immune activation drives inflammation, even if it is not an initial disease trigger. For example, intra‐articular monocyte/macrophage TLR activation may perpetuate joint inflammation in rheumatoid arthritis 40. Therapeutic inhibition of TLRs and their downstream signalling has been proposed for both SLE and rheumatoid arthritis: indirect modulation by Leukadherin‐1 may be a realistic alternative strategy 41.

There are caveats. For example, the small induction of IL‐12 by Leukadherin‐1‐treated monocytes runs contrary to the anti‐inflammatory trend: whether this low concentration is of any functional significance needs to be determined. Further evaluation of Leukadherin‐1 in other cell types will be important because we have already seen a proposed role for CR3 immune signalling not only in monocytes, but in B cells and dendritic cells as well 6, 7. Despite the caveats, initial reports from animal studies suggest efficacy in a range of inflammatory models, including vascular injury, chemical peritonitis and immune complex nephritis with low 60‐day toxicity 17, 18. Our data extend this to demonstrate encouraging anti‐inflammatory effects in a broad range of human *ex‐vivo* cell assays. There is considerable scientific rationale to support the hypothesis that CR3 activation is of therapeutic potential in a range of inflammatory disease situations. We would support continued evaluation in relevant animal models and the accrual of longer‐term safety data, with a view to future trials in human disease.

## Disclosure

The authors declare that they have no competing interests.

## Supporting information

Additional Supporting information may be found in the online version of this article at the publisher's web‐site:

**Fig. S1**. The effect of Leukadherin‐1 (LA1) on inflammatory cytokine secretion by Fc‐receptor engaged natural killer (NK) cells. Cells were pretreated with LA1 and stimulated using immunoglobulin (Ig)G‐coated polystyrene particles (*n* = 12). Dimethylsulphoxide (DMSO) provides the vector control for LA1. Data presented are mean ± standard error of the supernatant cytokine concentration at 24 h. Statistical testing by two‐tailed paired *t*‐test.
**Fig. S2**. The effect of Leukadherin‐1 (LA1) on Syk phosphorylation. Cells were incubated with either LA1 or dimethylsulphoxide (DMSO) for 30 min (*n* = 12). DMSO provides the vector control for LA1. Cells were permeabalized and stained with Alexa647 anti‐pSyk. Data presented are mean ± standard error of the median fluorescence of pSyk. Statistical testing by two‐tailed paired *t*‐test.
**Fig. S3**. The effect of interleukin (IL)‐10 receptor‐blocking antibodies on IL‐12+IL‐15‐ and IL‐12+IL‐18‐stimulated natural killer (NK) cell cytokine secretion. NK cells were incubated with anti‐CD210 antibody or isotype control together with IL‐12+IL‐15 (top panel) or IL‐12–IL‐18 (bottom panel). Data presented are mean ± standard error of the supernatant cytokine concentration at 24 h. Statistical testing by two‐tailed paired *t*‐test.
**Fig. S4**. The effect of Leukadherin‐1 (LA1) on in p38, extracellular‐regulated kinase (ERK), signal transducer and activator of transcription (STAT)‐3 phosphorylation. (a) Heat map (log_2_ median fold‐change) of effects of LA1 pretreatment on phosphorylated STAT (pSTAT)‐43, p‐p38, pERK and pSTAT‐5 following natural killer (NK) cell stimulation with interleukin (IL)‐12+IL‐18 (top panel) and IL‐12 + IL‐15 (lower panel) in total NK cells (left panel) and CD56^hi^ (right panel) across multiple time points. Three to nine samples per time‐point.
**Table S1**. The effect of Syk blockade on cytokine secretion by stimulated, Leukadherin‐1 treated natural killer (NK) cells
