# Supplementary material for: The complement receptor 3 (CD11b/CD18) agonist Leukadherin‐1 suppresses human innate inflammatory signalling
Source: Clin Exp Immunol. 2016 Jul 26;185(3):361–71. doi: 10.1111/cei.12803 (PMC4991522; doi:10.1111/cei.12803)

SUPPLEMENTARY MATERIAL

Supplementary Figure 1: The effect of Leukadherin-1 on inflammatory cytokine secretion by Fc-receptor engaged NK cells.

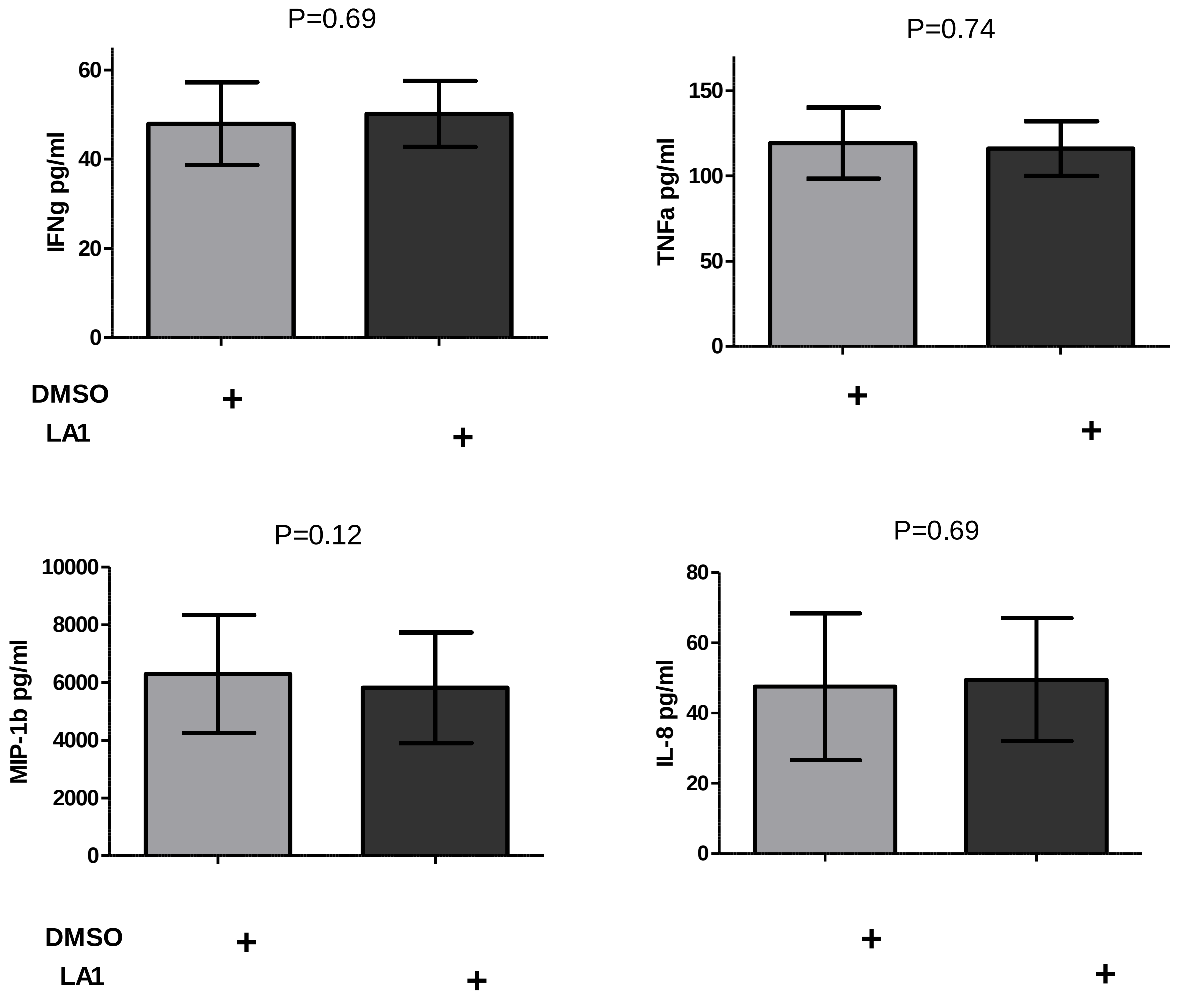

Cells were pretreated with Leukadherin-1 (LA1) and stimulated using IgG coated polystyrene particles (N=12). DMSO provides the vector control for Leukadherin-1. Data presented are mean +/- standard error of the supernatant cytokine concentration at 24 hours. Statistical testing by 2-tailed paired t-test.

Supplementary Table 1: The effect of Syk blockade on cytokine secretion by stimulated, Leukadherin-1 treated NK cells.

	LA1 + IL-12 + IL-15 stimulated (N=6)		LA1 + IL-12 + IL-18 stimulated (N=5)	
Secreted Cytokine	Mean Change with syk inhibitor (pg/ml)	p-value	Mean Change with syk inhibitor (pg/ml)	p-value
IFN-γ	-1170 (400)	0.043	-264 (2594)	0.92
TNF	-43 (17)	0.052	-59 (18)	0.0046
MIP-1β	-6810 (1050)	0.0029	-1884 (474)	0.029
IL-8	-106 (57)	0.12	-21 (6.7)	0.033

The mean (standard error) change in cytokine secretion consequent upon anti-Syk treatment, when compared with the reduced cytokine secretion already observed (figure 1) following treatment with Leukadherin-1 (LA1). Leukadherin-1 + IL-12 + IL-15 stimulation (left) or Leukadherin-1 + IL-12 + IL-18 stimulation (right). Statistics by 2-tailed paired t test.

Supplementary Figure 2: The effect of Leukadherin-1 on Syk phosphorylation

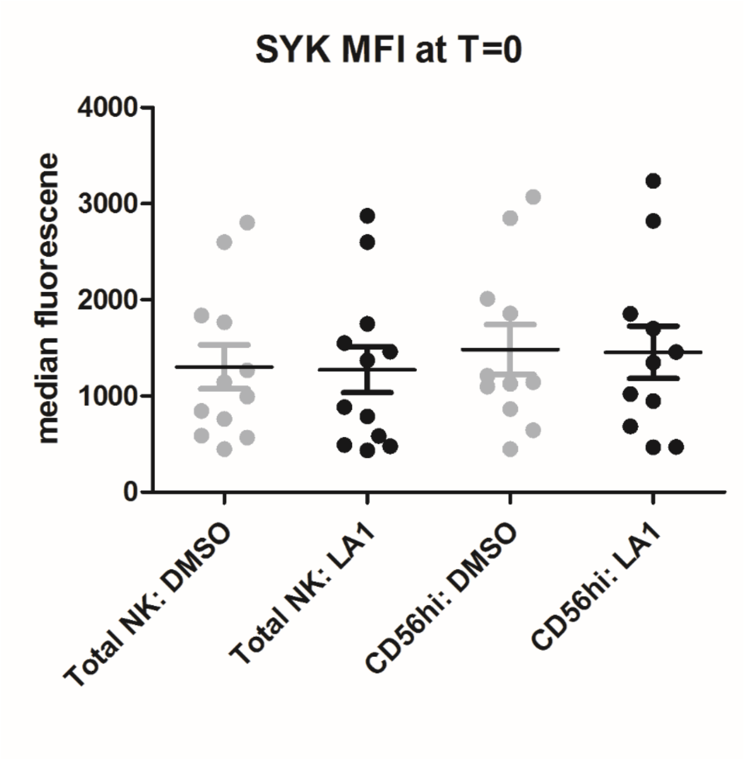

Cells were incubated with either Leukadherin-1 (LA1) or DMSO for 30 minutes (N=12). DMSO provides the vector control for Leukadherin-1. Cells were permeabalised and stained with Alexa647 anti-pSyk. Data presented are mean +/- standard error of the median fluorescence of pSyk. Statistical testing by 2-tailed paired t-test.

Supplementary Figure 3: The effect of IL-10 receptor blocking antibodies on IL-12+IL-15- and IL-12+IL-18-stimulated NK cell cytokine secretion

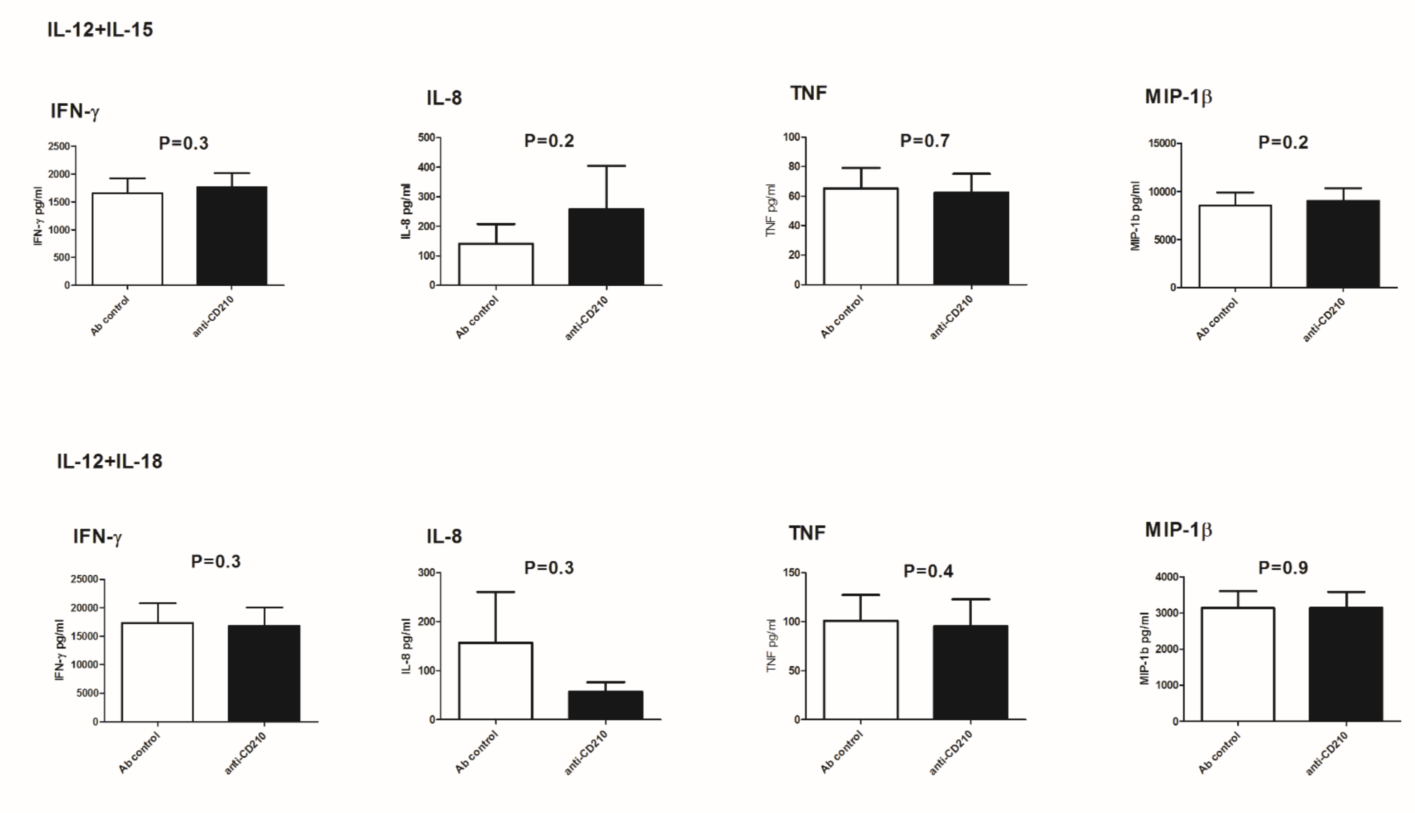

NK cells were incubated with anti-CD210 antibody or isotype control together with IL-12+IL-15 (top panel) or IL12-IL-18 (bottom panel). Data presented are mean +/- standard error of the supernatant cytokine concentration at 24 hours. Statistical testing by 2-tailed paired t-test.

Supplementary Figure 4: The effect of Leukadherin-1 on in p38, ERK, STAT3 phosphorylation

A) Heat map (log2 median fold-change) of effects of Leukadherin-1 pre-treatment on pSTAT43, p-p38, pERK and pSTAT5 following NK cell stimulation with IL-12+IL-18 (top panel) and IL-12+IL-15 (lower panel) in total NK cells (left panel) and CD56hi (right panel) across multiple time points. Three to nine samples per time point.

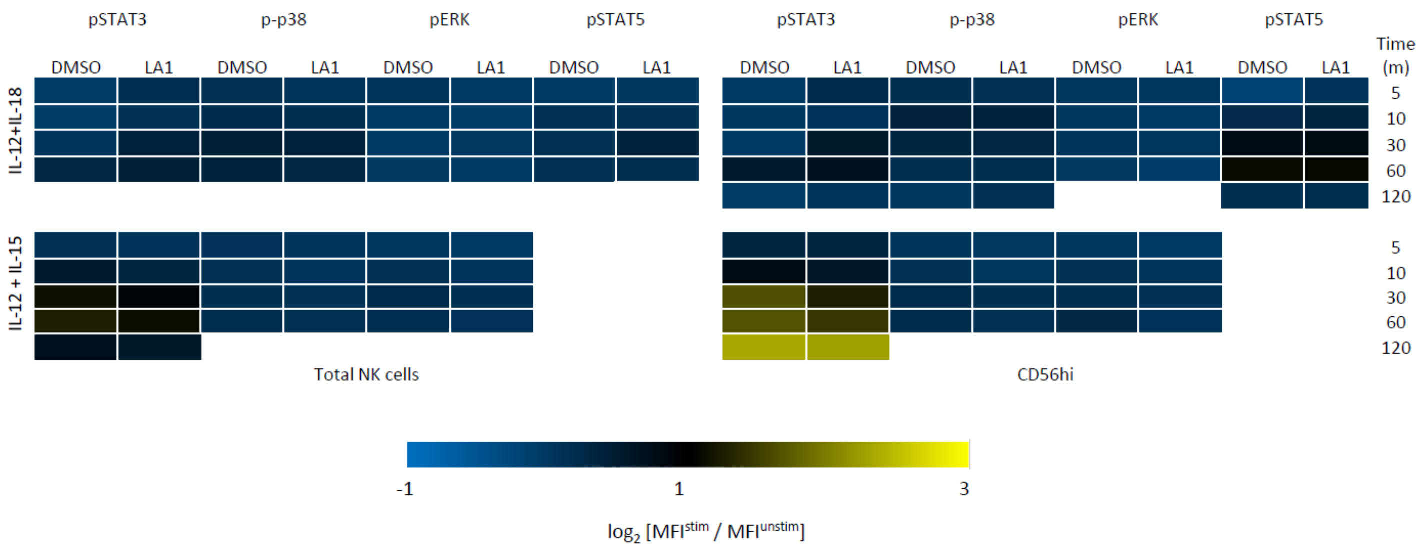

Supplement: Supplementary file 1 — Fig. S1. The effect of Leukadherin‐1 (LA1) on inflammatory cytokine secretion by Fc‐receptor engaged natural killer (NK) cells. Cells were pretreated with LA1 and stimulated using immunoglobulin (Ig)G‐coated polystyrene particles (n = 12). Dimethylsulphoxide (DMSO) provides the vector control for LA1. Data presented are mean ± standard error of the supernatant cytokine concentration at 24 h. Statistical testing by two‐tailed paired t‐test. Fig. S2. The effect of Leukadherin‐1 (LA1) on Syk phosphorylation. Cells were incubated with either LA1 or dimethylsulphoxide (DMSO) for 30 min (n = 12). DMSO provides the vector control for LA1. Cells were permeabalized and stained with Alexa647 anti‐pSyk. Data presented are mean ± standard error of the median fluorescence of pSyk. Statistical testing by two‐tailed paired t‐test. Fig. S3. The effect of interleukin (IL)‐10 receptor‐blocking antibodies on IL‐12+IL‐15‐ and IL‐12+IL‐18‐stimulated natural killer (NK) cell cytokine secretion. NK cells were incubated with anti‐CD210 antibody or isotype control together with IL‐12+IL‐15 (top panel) or IL‐12–IL‐18 (bottom panel). Data presented are mean ± standard error of the supernatant cytokine concentration at 24 h. Statistical testing by two‐tailed paired t‐test. Fig. S4. The effect of Leukadherin‐1 (LA1) on in p38, extracellular‐regulated kinase (ERK), signal transducer and activator of transcription (STAT)‐3 phosphorylation. (a) Heat map (log2 median fold‐change) of effects of LA1 pretreatment on phosphorylated STAT (pSTAT)‐43, p‐p38, pERK and pSTAT‐5 following natural killer (NK) cell stimulation with interleukin (IL)‐12+IL‐18 (top panel) and IL‐12 + IL‐15 (lower panel) in total NK cells (left panel) and CD56hi (right panel) across multiple time points. Three to nine samples per time‐point. Table S1. The effect of Syk blockade on cytokine secretion by stimulated, Leukadherin‐1 treated natural killer (NK) cells [file CEI-185-361-s001.pdf]
